# Use of a mHealth System to Improve Antenatal Care in Low and Lower-Middle Income Countries: Report on Patients and Healthcare Workers’ Acceptability in Tanzania

**DOI:** 10.3390/ijerph192215342

**Published:** 2022-11-20

**Authors:** Stefania Paduano, Federica Incerti, Lucia Borsari, Anne Caroline Benski, Alex Ernest, Ipyana Mwampagatwa, Athanase Lilungulu, Theresia Masoi, Annalisa Bargellini, Federica Stornelli, Giovanna Stancanelli, Paola Borella, Maria Angelica Rweyemamu

**Affiliations:** 1 Department of Biomedical, Metabolic and Neural Sciences, Section of Public Health, University of Modena and Reggio Emilia, 41125 Modena, Italy; 2 Department of Public Health, AUSL Modena, 41126 Modena, Italy; 3 Service d’Obstétrique-Département de la Femme, de l’Enfant et de l’Adolescent-Hôpitaux Universitaires de Genève, 1205 Genève, Switzerland; 4 Takemi Program in International Health, Harvard T.H. Chan School of Public Health, Boston, MA 02115, USA; 5 Department of Obstetrics and Gynecology, University of Dodoma, Dodoma 41218, Tanzania; 6 Cooperazione Paesi Emergenti, COPE NGO, Nyololo 51410, Tanzania; 7 Terre Innovative Healthcare S.R.L., 95126 Catania, Italy

**Keywords:** mobile health, antenatal care, pregnant women, health education, pregnancy

## Abstract

Antenatal care (ANC) is considered a cornerstone of reproductive health programmes, but many women face difficulties in accessing these services, particularly in some sub-Saharan African countries, such as Tanzania. This study aimed to test ANC visit acceptability using mHealth system PANDA (Pregnancy And Newborn Diagnostic Assessment) in the Mufindi district (Tanzania). We investigated the ANC visit acceptability of pregnant women and healthcare workers (HCWs) in an intervention area using the PANDA system compared with a control area. An ad hoc questionnaire was administered to pregnant women in an implementation area (*n* = 52) and in a control area (*n* = 46). In the implementation area, group interviews with 50 pregnant women were conducted and five HCWs evaluated ANC visits through a questionnaire. The implementation group was significantly more satisfied with the ANC visit compared with the control group. All the 52 women and the HCWs declared that PANDA icons were useful in understanding and remembering the provided information and the PANDA app was able to improve the ANC quality and to positively influence the relationship of HCWs and pregnant women. HCWs reported that the PANDA app was “easy-to-use” and “able to improve the adherence to ANC WHO recommendations”. In underserved areas, many pregnant women could benefit from the PANDA system increasing their access to high-quality ANC and overcoming language and/or literacy barriers.

## 1. Introduction

Globally, there were an estimated number of 295,000 maternal deaths in 2017, with an overall maternal mortality ratio (MMR) of 211 maternal deaths per 100,000 live births, out of which roughly two-thirds occurred in Sub-Saharan Africa [1]. In particular, the United Republic of Tanzania has one of the highest estimated numbers of maternal deaths, accounting for 524 deaths per 100,000 people. Several factors are linked to these deaths, including inadequate quality of services, limited ability to independently access health services, and direct complications related to childbirth such as postpartum haemorrhage or peri-partum sepsis. Most maternal deaths could potentially be avoided by ensuring better access to childbirth assistance and emergency obstetric care [2]. According to 2010–2016 national data, in the United Republic of Tanzania only 63.5% of births were assisted by skilled health personnel [3].

Stillbirths and neonatal deaths also present an important global health issue, mainly in low- and middle-income countries (LIMC). In 2019, Sub-Saharan Africa was the region with the highest stillbirth and under-five mortality rate worldwide, with 21.7 stillbirths per 1000 total births (seven times higher than in high-income settings) [4] and 76 under-five deaths per 1000 live births (10 times higher than in high-income countries). One-third of these deaths occur on the day of birth [5]. Most neonatal deaths occur as a result of infections, preterm birth complications, and intra-partum related adverse events. Evidence, therefore, suggests that focusing on the pre and post-partum period is essential to reduce the risk of death among newborns and their mothers [1,2,5].

Antenatal Care (ANC) represents the routine care provided by skilled health-care professionals to pregnant women in order to ensure the best health care conditions for both mothers and newborns and is considered a cornerstone of reproductive health programs [6]. The World Health Organization (WHO) recommends eight ANC contacts and four Post-Natal Care (PNC) visits [3,7,8,9]. Evidence suggests that ANC can improve maternal and neonatal health by providing health education and health promotion, and by preventing and managing potential health problems during pregnancy [6,10]. According to WHO guidelines, comprehensive ANC should comprise quality medical services, including the availability of screening tests, adequate treatments, and the provision of health education [10]. These aspects are not always available to pregnant women in LIMC, where there is a shortage of skilled health personnel. Moreover, individual perceptions of ANC, maternal education, past pregnancy, fear of pregnancy disclosure, and socio-cultural beliefs were reported as the key factors for late ANC attendance [11,12]. On the other hand, shortage of trained healthcare workers (HCWs), lack of spouse’s escort, and HCWs’ disrespect to pregnant women were the main barriers to early ANC attendance [12]. Hulsbergen et al. observed that disrespectful care was linked to factors which made the working circumstances of HCWs more difficult, such as resource shortages, low levels of integrated care, inadequate referral systems, and bad management [13].

Evidence increasingly shows the effectiveness of telemedicine services to support conventional healthcare, particularly among underserved communities, where the mobile health technologies (mHealth) can both facilitate quality data collection and improve ANC and PNC services [14,15] by providing HCWs with a defined path for carrying out a standardized visit in accordance with the international and national guidelines.

According to this evidence, the PANDA (Pregnancy And Newborn Diagnostic Assessment) utilizes mobile technology to facilitate the provision of high-quality, standardized ANC in underserved communities [16]. The system has been evaluated in terms of functionality and acceptability in two preliminary studies [10,16]. The first took place in the Refugees Camp of Mineo, Italy, and involved 250 pregnant migrants [16]. The second study took place in the Ambanja district, Madagascar, and looked at a cohort of 100 pregnant women [10]. The results of both studies demonstrated that PANDA is an efficient system for providing comprehensive and high-quality ANC and allows continuity of care in underserved communities. The process defined by the PANDA system makes it possible to standardize ANC visits and therefore carry out a complete visit without the risk of skipping important steps. Furthermore, the icon-based interface allows a better comprehension of the entire ANC visit process by pregnant women.

This study aimed to evaluate the acceptability of ANC visits using the PANDA system by the service recipients (pregnant women) as well as by the system users (healthcare workers or HCWs) in an area of the Mufindi district in Tanzania as compared to a control area.

## 2. Materials and Methods

### 2.1. The PANDA System

**Figure 1:**
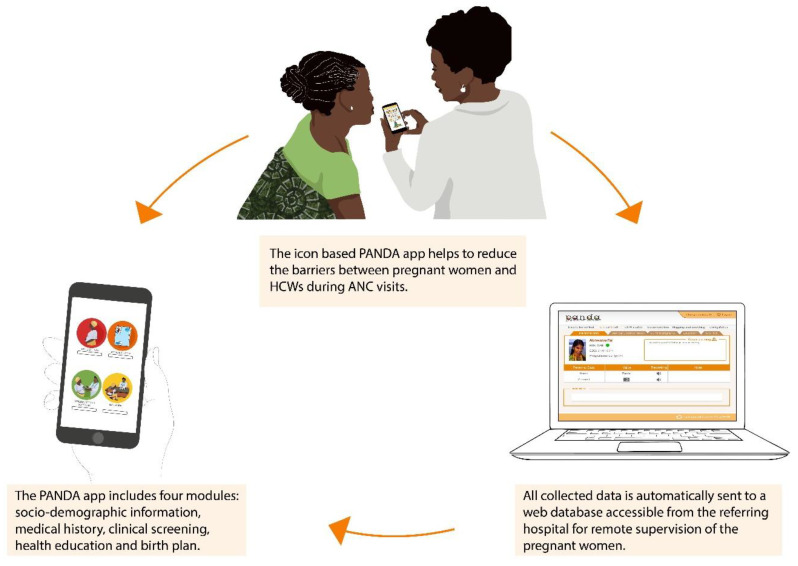
The PANDA system structure.

The structure of the PANDA system is shown in Figure 1. The core of the system resides in the icon-based Android application to collect socio-demographic information, medical history, and perform clinical screening. Moreover, the PANDA app includes an Education Module with information regarding hygiene, nutrition, risks during pregnancy, and child preparedness newborn care, according to the WHO recommendations [8]. The application uses various data-collection methods including photos, icon selection, free writing, and GPS. An alarm system alerts all registered risk conditions, allowing HCWs to promptly refer patients at risk. All collected data are automatically sent to a web database called Medical Unit accessible from the referring hospital, allowing remote supervision of the visits, bringing on-site HCWs closer to the referral hospital in order to provide women with a better ANC service. The PANDA system also works offline and in case of internet connection issues, as soon as the internet coverage becomes available, synchronization with the medical unit takes place.

### 2.2. Study Setting and Study Design

**Figure 2:**
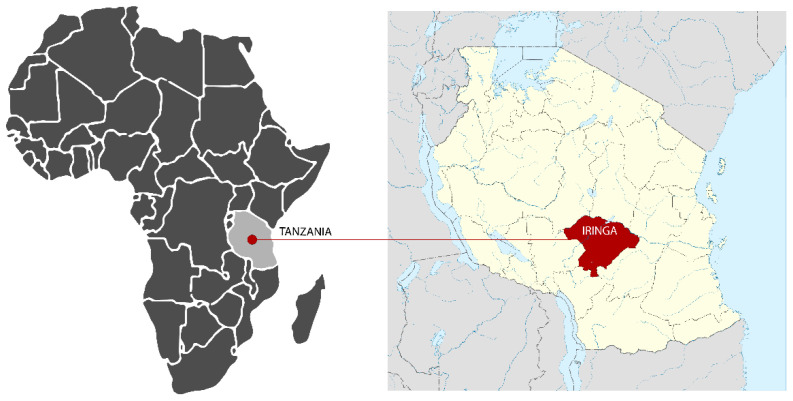
Iringa region, Tanzania.

To evaluate patients’ and HCWs’ acceptability of the PANDA system, an investigation was carried out in the Mufindi district in Iringa Region, Tanzania (Figure 2). The implementation site was located in the Nyololo and Maduma villages, while the control site was located in Mbalamaziwa and Nyanyembe. The region of Iringa is in a highland plateau and the villages included in the survey are all located at an altitude of over a thousand meters. The villages are about half an hour’s drive from each other and two hours from Iringa, the regional capital. During a two-month period, questionnaires and group interviews were performed in the implementation site, where the PANDA system was used to perform ANC visits, compared with the control area, where ANC visits were performed without the use of the PANDA system.

This study is part of a large non-randomized intervention trial conducted from June 2019 to September 2020, carried out to compare the quality of ANC visits by using the PANDA system in the intervention site with standard visits performed according to the protocols already in use in the control site.

The selected sites were similar in terms of personnel and demand, serving a total population of about 28,000 individuals. Each of the two health centers have about 250 childbirths per year, no caesarean sections were performed and a referral procedure was in place in both centers for all pregnant women in need of second aid medical intervention. The staff in both intervention and control sites were trained on ANC guidelines, and only HCWs in the intervention site were also trained on the use of the PANDA system.

The mHealth utilized in the implementation site combines existing smartphone technologies in one easy-to-use, cheap, robust, and portable diagnostic tool connected remotely to a centralized database.

### 2.3. Study Population and Data Collection

A total of 98 women took part in the questionnaire about the acceptability of ANC visits (52 women in the implementation area, and 46 in the control area) during the last two months of study. All pregnant women were eligible with signed informed consent and regardless of age, gestational age, religion, or ethnicity.

In the implementation area, data were collected using the PANDA application during the ANC visits. In the control area the data were collected periodically from clinical registers.

The questionnaire used was modified from the standardized questionnaire proposed by the WHO to assess the perceived quality of ANC and it had two sections [16,17]. The first section concerned of items on the visit such as the duration, the availability of screening tests, and drugs for recommended prophylaxis during pregnancy. The second section investigated the completeness of health education with information on how to adopt healthy behavior, explanation of screening tests, a description of the risks during pregnancy, labor, as well as recommendations on birth preparedness and new-born care. In the intervention site, additional items were included to assess the women’s perception about PANDA visits and about the usefulness of the icons in understanding and remembering the information provided. Finally, the usefulness of the PANDA app in improving ANC quality and in influencing the relationship between HCWs and pregnant women was investigated.

Moreover, 5 group interviews with a total of 50 women were conducted in the implementation area. The group interviews were held at the implementation site and included women who had participated in ANC visits with the PANDA system and had already given birth. A grid of open questions was prepared ad hoc. A facilitator was appointed to conduct the guided interviews, stimulating participation by asking the questions clearly and simply in local language. During the group interviews, an observer took note of what was said and noted down non-verbal cues that emerged during the discussion. The five group interviews (about 10 women per group) lasted about 2 h and firstly explored the family’s attitude towards woman’s participation in antenatal visits and woman’s autonomy over family planning. Then, women’s knowledge about risks during pregnancy, delivery, and postpartum was investigated as well as the reasons behind the choice to give birth at home and the importance of skilled personnel for the birth. Finally, they were asked about their feelings during the ANC visit whether they felt welcomed and reassured by the HCWs or felt confused and scared during the visits. All individual interviews and group interviews were conducted by trained staff in Swahili language.

In the implementation site, a closed-ended questionnaire was also administered to HCWs in order to assess their acceptability of the PANDA system. The questionnaire consisted of two parts. The first section included some questions about time spent in ANC visits, information provided on health education, and on how to recognize and to proceed in case of problems during pregnancy and childcare. The Second part collected information on the usefulness of PANDA icons in understanding and remembering information and on the efficacy of the PANDA app in improving ANC quality, adherence to WHO recommendations, the relationship between patients and HCWs, and patients’ satisfaction.

### 2.4. Data Analysis

Stata Statistical Software Release 15 (StataCorp., College Station, TX, USA) was used for data analysis. Categorical data were reported as frequency. Comparisons between groups were assessed by Chi-square test.

## 3. Results

This study involved 52 pregnant women aged between 17 and 40 years (mean age: 25.8) recruited in the implementation area and 46 pregnant women aged between 18 and 40 years (mean age: 26.6) in the control area. Most of the participants (78.9% in the intervention area vs. 73.9% in the control area) were multigravida and 53.8% in the intervention area vs. 52.2% in the control area reported two to five previous childbirths. No difference was found about obstetric history between the intervention and control areas.

### 3.1. Women’ Acceptability

Data collected from the questionnaire administered to the two subgroups of pregnant women showed a significantly higher degree of acceptability in the implementation area than in the control area (Table 1).

In the implementation area, almost all pregnant women (96.2%) considered the time spent with HCWs was adequate during ANC visits with a significant difference compared with the women in the control area, where the majority of women (78.3%) reported that the duration of the visit was less than expected. Significant differences between the two groups were also found regarding the health information provided to them regarding danger signs, labor, and breastfeeding: almost all women in the control group reported that it was “as much as they expected”, while the majority of women in the implementation area considered it “more than expected”. Women in the implementation area were significantly more likely to return to ANC in a future pregnancy and to recommend ANC to relatives or friends than in the control group. The intervention group was significantly more satisfied with the overall ANC visit in comparison with the control group.

In the implementation site, all the pregnant women declared that PANDA icons were useful in understanding and remembering information provided during ANC visit; the PANDA app was able to improve ANC quality and to positively influence the relationship between HCWs and pregnant women.

During the group interviews, it emerged that many women had difficulty following the recommendations of the health personnel because domestic duties were often in conflict with health recommendations. A common concern was to share certain symptoms for fear of learning of a miscarriage, as this is perceived by the community as a consequence of something done wrong by the mother. The staff was supportive in overcoming this problem by using PANDA tool to facilitate comprehension. In general, women were satisfied with the service provided and the attention received, also thanks to a greater proximity, even physical, during the entire visit. Therefore, regarding the relationship with HCWs, women felt less intimidated and more encouraged to ask them for counselling. The icon-based application facilitated the comprehension of all ANC steps by overcoming literacy and language issues, even if some women had difficulties in understanding the usefulness of the screening tests. All women agreed that HCWs adequately prepared them, especially during visits closer to the expected date of birth, thanks to the app module of health education and birth preparedness. Finally, the women expressed their wish that the Tanzanian ministry use the PANDA system on a large scale.

### 3.2. Acceptability of HCWs

A total of five midwives filled out a self-administered questionnaire about the PANDA system’s acceptability in the implementation area. Three out of five found that the number of ANC visits was “about right”, while the remaining considered it “more than necessary”. They evaluated that the time spent in PANDA visits was “about right”. All midwives reported that all the important information on health education (e.g., nutrition, personal hygiene, tests during the pregnancy, labor, delivery, breastfeeding, and where to give birth) was provided during the visits, except for items about working during pregnancy and family planning. Two out of five responders reported that no information was provided to the pregnant women on how to recognize premature contractions and to proceed in case of rupture of membranes and premature contractions, whereas at least four out of five midwives revealed that the other information about recognition and management of serious problems during pregnancy (e.g., hemorrhage, dizziness and fainting, and high fever) and child care (e.g., fever or low temperature, convulsions, navel infections, inability to breastfeed, and breathing difficulties) was provided to the women. All responders scored PANDA visits as “very good” and “to recommend to relative or friend” and they perceived satisfaction among patients. The PANDA app was evaluated as “easy-to-use”, “able to influence the relationship between HCWs and pregnant women”, and “able to improve ANC quality and the adherence to ANC WHO recommendations”. PANDA icons were assessed as “useful for the women to understand and to remember information provided during the visits”.

## 4. Discussion

This study investigated pregnant women’s and HCWs’ acceptability of ANC visits realized through the PANDA system. Antenatal care is a key element in ensuring that each woman’s pregnancy could be a positive experience [6].

Telemedicine and mHealth can play an important role in raising the quality of service, and it is key to measure the quality level perceived by patients and HCWs [18,19]. Effective communication is an important factor in improving subjects’ acceptability and satisfaction regarding maternity and newborn care. Providing clear and available information is the first step towards satisfaction and awareness of pregnant women [20,21]. Nair et al. reported that a passive flow of information from HCWs to pregnant women is not effective in increasing patients’ knowledge and awareness about their health condition to allow shared decision-making [20].

In this study, pregnant women who had received ANC visits with the PANDA system showed a high level of satisfaction. Despite the longer time spent for a visit by using the PANDA app and consequently longer wait times, in the implementation site almost all women considered duration of the visit as optimal, compared with only 22% of the control group. Indeed, the longer time spent for a PANDA visit is a direct measure of outcome: the sequential screens of the application oblige the operator to perform a complete examination, avoiding the omission of any data collection or screening examinations.

Women reported that the health information received during the PANDA ANC visit was complete and clear, thanks to the intuitive and easy-to-understand graphic interface, which facilitated the communication between HCWs and patients, overcoming most language and literacy barriers. Thus, PANDA icons positively influenced their relationship, creating a greater empathy between the HCWs and the women. All the 52 pregnant women interviewed declared that PANDA icons were also useful in remembering health education. Information about family planning was the only lacking issue. This can be related to a presidential note, issued during the study, which called for a freeze on all family planning activities, resulting in a reduction in this specific health education activity.

In general, the interviewed women showed great appreciation of the system, as evidenced by the high percentage who responded positively to the intention to return for a subsequent pregnancy and to recommend to family and friends the ANC service provided by the PANDA system. Moreover, the number of women who had at least four antenatal visits was higher in the intervention group, meaning that the system was able to communicate the importance of WHO and national recommendations to carry out the appropriate number of visits.

Although generalization is not possible, from the items in which all five HCWs agreed, we can infer that their attitude towards the mHealth tool was in general very positive; all of them considered the PANDA system as an easy-to-use and very supportive device in improving the relationship with women. The smartphone, unlike other technical tools such as the personal computer, does not create a barrier but rather a greater proximity, even physical. The visit with the PANDA was carried out with HWCs and women sitting next to each other to facilitate the viewing of images in the smartphone, useful to overcome possible literacy and language issues. This mode of ANC visit provision has a positive impact on learning and in general on mutual trust, reducing the barriers usually created by physical distance during standard visits. Finally, the longer duration of the visits was not perceived by the HCWs as an extra workload, but rather as an improvement in the quality of the service offered with greater adherence to national and international guidelines.

The PANDA system enables HCWs to provide a service according to the national and international recommendations, due to a defined path that supports HCWs in carrying out a standardized visit reducing HCW-associated issues [22]. Moreover, we think that using the PANDA system makes HCWs aware that the data will be automatically be sent and reported to the referral hospital. A key factor to improve the quality of the ANC services is the support of the medical unit to the on-site HCWs [13]. The easy-to-use icon-based interface allows task shifting towards less-trained HCWs without compromising the quality of care [23,24]. Indeed, an alert signal system included in the app suggests the woman’s referral in case of abnormal values. Moreover, the supervision of the medical unit within the referral hospital is a further guarantee of proper follow-up of risk conditions for the woman and/or her unborn child. These aspects are overall relevant in low-resource and underserved areas where the shortage of trained personnel is a big concern [10].

## 5. Conclusions

The PANDA system provides a promising solution to improve the quality and utilization of ANC visits and to overcome language and/or literacy barriers. In low-resource and underserved areas, pregnant women can benefit from this system. Furthermore, this mHealth system is useful for public health policymakers, as it allows the automatic collection socio-demographic and clinical data. However, it is only relevant for public health policy making if such a system is implemented nationally in a standardized way, not only ensuring comprehensive data collection but also equity in providing standardized quality care.

## Figures and Tables

**Table 1:** ANC acceptability assessment of pregnant women by area (implementation area (*n* = 52) and control area (*n* = 46)).

Items	Questions	Pregnant Women		*p*-Value
		Implementation Site	Control Site	
		*n* (%) *	*n* (%) *	
Duration of the visit	a lot more time	2 (3.8)	0 (0.0)	<0.001
	a little more time	0 (0.0)	36 (78.3)	
	right time	50 (96.2)	10 (21.7)	
Health education during the ANC	No	0 (0.0)	0 (0.0)	0.125
	sometimes	0 (0.0)	2 (4.4)	
	yes	52 (100.0)	43 (95.6)	
Availability medicines prescribed by HCWs	no	0 (0.0)	0 (0.0)	0.125
	sometimes	0 (0.0)	2 (4.4)	
	yes	52 (100.0)	43 (95.6)	
Availability screening tests	no	0 (0.0)	0 (0.0)	0.059
	sometimes	0 (0.0)	3 (6.7)	
	yes	52 (100.0)	42 (93.3)	
Information regarding the woman’s health	no information	0 (0.0)	0 (0.0)	<0.001
	not enough	0 (0.0)	0 (0.0)	
	as much as they expected	2 (3.8)	45 (97.8)	
	more than expected	50 (96.2)	1 (2.2)	
Information regarding Screening during ANC	no information	0 (0.0)	0 (0.0)	<0.001
	not enough	0 (0.0)	0 (0.0)	
	as much as they wanted	1 (1.9)	45 (97.8)	
	more than expected	51 (98.1)	1 (2.2)	
Information regarding danger signs during pregnancy	no information	0 (0.0)	0 (0.0)	<0.001
	not enough	0 (0.0)	0 (0.0)	
	as much as they expected	2 (3.8)	45 (97.8)	
	more than expected	50 (96.2)	1 (2.2)	
Information regarding labor	no information	0 (0.0)	0 (0.0)	<0.001
	not enough	0 (0.0)	0 (0.0)	
	as much as they expected	0 (0.0)	45 (97.8)	
	more than expected	52 (100.0)	1 (2.2)	
Information regarding labor breastfeeding	no information	0 (0.0)	0 (0.0)	<0.001
	not enough	0 (0.0)	0 (0.0)	
	as much as they expected	0 (0.0)	46 (100.0)	
	more than expected	52 (100.0)	0 (0.0)	
Overall satisfaction of the ANC visit	absolutely not satisfied	0 (0.0)	0 (0.0)	<0.001
	not so satisfied	0 (0.0)	0 (0.0)	
	quite satisfied	0 (0.0)	9 (19.6)	
	very satisfied	52 (100.0)	37 (80.4)	
If they become pregnant again, will they come back for an ANC visit?	absolutely not	0 (0.0)	0 (0.0)	<0.001
	probably not	0 (0.0)	0 (0.0)	
	probably yes	0 (0.0)	11 (23.9)	
	absolutely yes	52 (100.0)	35 (76.1)	
Recommendation to a relative or a friend for ANC	absolutely not	1 (1.9)	0 (0.0)	<0.001
	probably not	0 (0.0)	0 (0.0)	
	probably yes	0 (0.0)	11 (23.9)	
	absolutely yes	51 (98.1)	35 (76.1)	

* The percentages were calculated excluding missing values.

## Data Availability

The data presented in this study are available on reasonable request from the corresponding author. The data are not publicly available due to privacy and legal restrictions.
